# Minocycline reduces intracerebral hemorrhage–induced white matter injury in piglets

**DOI:** 10.1111/cns.13220

**Published:** 2019-09-26

**Authors:** Heng Yang, Xin‐Jie Gao, Yan‐Jiang Li, Jia‐Bin Su, Tong‐Zhou E, Xin Zhang, Wei Ni, Yu‐Xiang Gu

**Affiliations:** ^1^ Department of Neurosurgery Huashan Hospital Fudan University Shanghai China

**Keywords:** intracerebral hemorrhage, minocycline, mitogen‐activated protein kinases, transforming growth factor‐β, white matter injury

## Abstract

**Aims:**

White matter (WM) injury after intracerebral hemorrhage (ICH) results in poor or even fatal outcomes. As an anti‐inflammatory drug, minocycline has been considered a promising choice to treat brain injury after ICH. However, whether minocycline can reduce WM injury after ICH is still controversial. In the present study, we investigate the effect and underlying mechanism of minocycline on WM injury after ICH.

**Methods:**

An ICH model was induced by an injection of autologous blood into the right frontal lobe of piglets. First, transcriptional analysis was performed at day 1 or 3 to investigate the dynamic changes in neuroinflammatory gene expression in WM after ICH. Second, ICH piglets were treated either with minocycline or with vehicle alone. All piglets then underwent magnetic resonance imaging to measure brain swelling. Brain tissue was used for real‐time polymerase chain reaction (RT‐PCR), immunohistochemistry, Western blot, and electron microscopy.

**Results:**

Transcriptional analysis demonstrated that transforming growth factor‐β (TGF‐β)/mitogen‐activated protein kinase (MAPK) signaling is associated with microglia/macrophage‐mediated inflammation activation after ICH and is then involved in WM injury after ICH in piglets. Minocycline treatment results in less ICH‐induced brain swelling, fewer neurological deficits, and less WM injury in comparison with the vehicle alone. In addition, minocycline reduces microglial activation and alleviates demyelination in white matter after ICH. Finally, we found that minocycline attenuates WM injury by increasing the expression of TGF‐β and suppressing MAPK activation after ICH.

**Conclusion:**

These results indicate that TGF‐β–mediated MAPK signaling contributes to WM injury after ICH, which can be altered by minocycline treatment.

## 1 BACKGROUND

Intracerebral hemorrhage (ICH) often results in brain injury, which contributes to the high morbidity and mortality of this subtype of stroke.^1^, ^2^, ^3^ The complex tissue pathology resulting from ICH involves both gray matter and white matter (WM). White matter fibers, including the corpus callosum, internal capsule, anterior commissure, and striatum bundles, are highly vulnerable to additional injury in ICH patients. Thus, WM injury reflects the brain's vulnerability to further insult and predicts poor outcomes after ICH.^4^, ^5^, ^6^ Our previous work has shown that ICH‐induced iron toxicity causes WM injury through c‐Jun N‐terminal kinase (JNK) and receptor‐interacting protein kinase 1 (RIPK1) activation.^7^ Because of white matter's critical role in neurotransmission, WM injury may also lead to severe sensorimotor dysfunction, neurobehavioral impairment, and cognitive disorders.^8^, ^9^ Therefore, protecting against WM injury after ICH is a pressing concern.

Transforming growth factor‐β (TGF‐β) is a ubiquitous anti‐inflammatory cytokine that is active in a variety of cell types. It is minimally expressed with latent isoforms in normal brain, but its expression increases strongly after several types of brain insult, including ischemia, trauma, and hemorrhage. TGF‐β modulates microglia‐mediated neuroinflammation after ICH, promotes functional recovery,^10^ and plays a pivotal role in progressive WM injury.^11^ It can be involved in the propagation of WM injury after ischemic stroke through the development of microangiopathy. Enhancing production of TGF‐β confers neuroprotection by alleviating WM injury.

Minocycline is a Food and Drug Administration (FDA)‐approved highly lipophilic antibiotic with the ability to penetrate the brain‐blood barrier (BBB), originally developed to treat meningitis. In neurovascular studies, minocycline shows anti‐inflammatory and neuroprotective effects in a rodent model of ICH and cerebral ischemia.^12^, ^13^ Our previous studies have demonstrated that minocycline is able to inhibit the inflammatory response to brain injury and reduce neuronal apoptosis, brain edema, and BBB disruption by iron chelation.^14^, ^15^ Although ongoing clinical trials suggest that minocycline may be a neurovascular protective agent in humans, the underlying mechanisms remain to be elucidated.^16^, ^17^ Previous studies have indicated that minocycline significantly reduces neuroinflammatory WM injury induced by hypoxia and alleviates WM‐related cognitive impairment after chronic hypoperfusion through its robust effects on oligodendrocyte progenitor cells.^18^, ^19^ We hypothesize that minocycline can reduce neuroinflammation and then alleviate WM injury and improve behavioral impairment after ICH.

In the present study, experimental ICH piglets were used to evaluate the beneficial efficacy of minocycline in treating WM injury after ICH. Intracerebral hemorrhage models have been established in many species, including rodent, cat, dog, rabbit, and piglet.^20^ We chose the piglet as it is a large mammal exhibiting close similarity to humans with a higher WM concentration in piglets' brains compared to other potential model species.^21^, ^22^

## 2 METHODS

### 2.1 Animal preparation and intracerebral infusion

The 56 male piglets (15‐25 kg) used in this study were obtained from the Experimental Animal Center of Shanghai Jiaotong University. Animal use protocols were approved by the Animal Ethics Committee of Fudan University. Two piglets died during initial anesthesia and were excluded from the study. The ICH models were generated as previously described.^23^, ^24^ Before the operation, the animals were acclimated to the surroundings for at least 1 week. The animals were sedated with ketamine (15‐20 mg/kg, IM) and diazepam (5‐10 mg/kg, IM) for induction of anesthesia and endotracheal intubation, and 5% pentobarbital sodium was used to maintain anesthesia during the surgical procedures. Body temperature was maintained at 37.5 ± 0.5°C by a heating pad. The right femoral artery was catheterized with a polyethylene catheter (PE‐160) to obtain blood for injection and monitor arterial blood pressure and blood gases. A cranial burr hole (1.5 mm) was then drilled 11 mm to the right of the sagittal suture and 11 mm anterior to the coronal suture. An 18.5‐mm‐long 18‐gauge sterile plastic catheter was placed stereotaxically into the center of the right frontal cerebral white matter at the level of the caudate nucleus. Autologous arterial blood of 1 mL of was infused for 10 minutes with an infusion pump. Then, another 1.5 mL of blood was injected for 10 minutes after 5 minutes.^24^ Sham piglets underwent the same procedure without blood infusion.

### 2.2 Experimental groups

The experiment was divided into two parts. In the first part, pigs (n = 8) had a right frontal injection of autologous blood. Pigs were euthanized at days 1 and 3 for transcriptional analysis (n = 4 per group per day). Sham‐operated pigs were used as controls (n = 4). In the second part, pigs had a right frontal injection of autologous blood and were treated with minocycline (4 mg/kg, intramuscularly, at 2 hours after ICH and then 2 mg/kg, intramuscularly, every 12 hours for 3 days) or vehicle alone. Control animals underwent only a needle insertion. The pigs were euthanized three and 14 days after surgery for RT‐PCR (n = 4 per group, day 3), immunohistochemistry (n = 3 per group per day), Western blot assay (n = 4 per group, day 3), and electron microscopy (n = 3 per group, day 14).

### 2.3 Transcriptional analysis

Transcriptional analysis was performed as described previously.^10^ In brief, gene ontology (GO) enrichment analysis, which describes gene properties, was performed, followed by mapping of genes to Kyoto Encyclopedia of Genes and Genomes (KEGG) pathways to identify genes potentially expressed after ICH. Heatmaps were used to demonstrate the dynamic changes in target gene expression after ICH. Pathway‐pathway analysis was then performed to identify potential interaction networks using Cytoscape 3.4.0 (Agilent and IBS).

### 2.4 Magnetic resonance imaging and brain swelling measurement

MRI scanning was performed immediately postoperation, and at days 3 and 14 postoperation. The animals were sedated with ketamine (15‐20 mg/kg, IM) and diazepam (5‐10 mg/kg, IM) for induction of anesthesia and endotracheal intubation. Then, 5% pentobarbital sodium was used to maintain anesthesia throughout MRI examination. MRI scanning was performed in a 3.0‐T Siemens AG MR scanner (183‐mm horizontal bore; Varian) at the Department of Radiology, Huashan Hospital, Fudan University. The imaging protocol for all the piglets included T2 fast spin‐echo sequence using a view field of 35 mm × 35 mm and 17 coronal slices (1.0 mm thickness). Image analysis was performed blind using ImageJ (National Institutes of Health, Bethesda, MD, USA) by two observers. The brain swelling rate is calculated as (ipsilateral cerebral hemisphere volume − contralateral cerebral hemisphere volume)/ipsilateral cerebral hemisphere volume × 100%. The quantification data of brain water content was showed in Table S1.

### 2.5 Behavioral test

The neurological status of each animal was evaluated according to the neurological grading score, with modifications (Table S2).^25^ Total neurological deficit included scores generated from a 25‐point scale that assessed appetite (0‐4), standing position (0‐5), head position (0‐2), utterance (0‐2), gait (0‐3), motor function (forelimbs/hind limbs; 0‐4), and facial paresis (0‐1) at 2 hours before surgery and at days 1, 3, 5, 7, and 14 after surgery. A total score of 25 indicates maximum impairment (comatose or dead pigs), whereas 0 denotes complete normality. The tests were conducted blind on the three groups by an observer.

### 2.6 Immunofluorescence staining

Paraffin‐embedded brains were cut into 10‐μm‐thick sections. The sections were deparaffinized in xylene and rehydrated in a graded series of alcohol dilutions. Antigen retrieval was performed by the microwave method using citrate buffer (10 mmol/L, pH 6.0). All sections were then treated with 0.3% hydrogen peroxide to neutralize endogenous peroxidases. The sections were then blocked with 3% bovine serum albumin (BSA) in 0.1% Triton X‐100 (v/v) for half an hour at room temperature and washed three times in 0.1 mol/L phosphate‐buffered saline (PBS, pH 7.4). The sections were incubated overnight with specific primary antibodies at 4°C. Following overnight incubation, the sections were rinsed with PBS and incubated at room temperature for 1 hour with secondary antibodies. The primary antibodies were monoclonal rabbit anti‐ionized calcium‐binding adaptor molecule 1 (Iba‐1, 1:1000; Wako), monoclonal rat anti‐myelin basic protein (MBP, 1:500; Abcam), monoclonal rabbit anti‐glial fibrillary acidic protein (GFAP, 1:300; Abcam), monoclonal rabbit anti‐CD31 (1:100; Abcam), monoclonal rabbit anti–TGF‐β (1:200; Abcam), polyclonal rabbit anti‐CD206 (1:200; Abcam), and polyclonal rabbit anti‐CD86 (1:300; Abcam). Secondary antibodies were Alexa Fluor 488 donkey anti‐rat IgG (1:1000; Jackson), Alexa Fluor 488 donkey anti‐rabbit IgG (1:1000; Jackson), and Alexa Fluor 594 donkey anti‐rabbit IgG (1:1000; Jackson).

### 2.7 Brain in situ freezing and sampling

Brains were frozen in situ by decanting liquid nitrogen into a 12‐oz bottomless foam cup affixed to the head with Dow Corning High‐Vacuum Grease (Dow Corning), as described previously.^26^ This process usually took ~1 hour, and the head was removed after injection of potassium chloride to stop the heart beating. The frozen head was then cut with a band saw into 5‐mm‐thick coronal sections. Tissues were sampled from the points of interest.

### 2.8 Western blotting

Western blot analysis was performed as previously described.^26^ White matter tissue adjacent to the hematoma was sampled and electrophoresed on SDS/PAGE gels. After transferring the protein to a PVDF membrane, corresponding proteins were probed with different antibodies and signals were detected using an ECL kit. The relative band densities were analyzed with ImageJ. The primary and secondary antibodies were polyclonal rabbit anti–interleukin‐1β (IL‐1β, 1:1000; Abcam), monoclonal rabbit anti–TGF‐β (1:1000; Abcam), monoclonal rabbit anti‐inducible nitric oxide synthase (iNOS, 1:1000; Abcam), monoclonal rabbit anti‐tumor necrosis factor α (TNF‐α, 1:1000; Abcam), monoclonal rabbit anti‐extracellular signal–regulated kinase (ERK, 1:1000; CST), monoclonal rabbit anti‐phosphorylated ERK (1:1000; CST), monoclonal rabbit anti‐p38 (1:1000; CST), monoclonal rabbit anti‐phosphorylated p38 (1:1000; CST), polyclonal rabbit anti–c‐Jun N‐terminal kinases (JNK, 1:500; SCB), and monoclonal mouse anti‐phosphorylated‐JNK (1:500; SCB).

### 2.9 Real‐time PCR

Real‐time polymerase chain reaction was performed as described previously.^27^ Briefly, total RNA was isolated with TRIzol reagent (Thermo Fisher Scientific); then, RNA was reverse‐transcribed into cDNA using the Superscript First‐Strand Synthesis System (Invitrogen). Real‐time polymerase chain reaction was performed using the Opticon2 Real‐Time PCR Detection System (Bio‐Rad) and SYBR PCR Master Mix (Invitrogen). Expression of glyceraldehyde 3‐phosphate dehydrogenase (GAPDH) mRNA served as an internal control. Gene expression at the mRNA level was normalized to GAPDH mRNA and expressed as fold change vs control. Primer sequences used for the inflammatory cytokines are listed in Table 1 (in pairs, sense and antisense).

**Table 1:** Primer sequences used for real‐time polymerase chain reaction

Gene	Forward primer	Reverse primer
GAPDH	5′‐GGAAGCTGTGGCGTGATGGC‐3′	5′‐TTCTCCAGGCGGCAGGTCAG‐3′
iNOS	5′‐GCTGAAGGCTCTCCACCTCCTC‐3′	5′‐GGTATCTTGTCATCGCTGTCATCTCC‐3′
TNF‐a	5′‐GCACTGAGAGCATGATCCGAGAC‐3′	5′‐CGACCAGGAGGAAGGAGAAGAGG‐3′
IL‐1β	5′‐TACAGGCTCGTGCAGGACTCAG‐3′	5′‐GGTGGTGCGGCTGGATTGC‐3′
TGF‐B1	5′‐AACCTACCCGACTGGTATC‐3′	5′‐CACAGCCGGACCTTTAAC‐3′
TGF‐BR2	5′‐TACCACGGATTTGTTCTCGAT‐3′	5′‐ACCCTTTCCTCTCCTTCATAA‐3′
BMP2	5′‐CAACAGAGGCAATGGAGC‐3′	5′‐TTGGCAGATGGCCTTGTAG‐3′
BMP7	5′‐GAGATGTTCGAGGCACAC‐3′	5′‐GTCTCATTCACTCAGCGGA‐3′
MYC	5′‐AACGTCAGCTTCACCAAC‐3′	5′‐AGAAATAAGGCTGCACCG‐3′
DUSP2	5′‐CTGGTTCCAGGAGGCTATC‐3′	5′‐CCTGCCTGGCAATGTACTA‐3′
PTPN7	5′‐CCTCTGCTTCCTCCAATG‐3′	5′‐GGTCAGCCACTAGCTTCAA‐3′
NFATC1	5′‐TGGTGGTGGACTCATATTCATC‐3′	5′‐GAAGAAGCTGAGAAGCTAAAGG‐3′
MAP4K1	5′‐TTTCATCCTGAACCGAAATGAC‐3′	5′‐GAGAGAGACATGAGGACATTG‐3′

### 2.10 Electron microscopy

Electron microscopy to access myelin and axon damage in the perihemorrhage area was performed as described previously.^28^ Briefly, piglets were perfused with saline, followed by ice‐cold 4% paraformaldehyde and 0.1% glutaraldehyde in 0.1 mol/L PBS (pH 7.4). White matter tissue in the perihematoma area was microdissected into 1‐mm blocks and fixed in 2% glutaraldehyde overnight. Then, the tissues were washed in 0.1 mol/L sodium cacodylate buffer (pH 7.4), postfixed in buffered osmium tetroxide for 1‐2 hours. Following serial dehydration in acetone, the tissue was embedded in epoxy resin. Sections of 60‐90 nm thickness were placed onto 200‐mesh grids, were stained with uranyl acetate and lead citrate, and were then examined with a JEOL JEM‐1230 transmission electron microscope. We calculated the G‐ratio (ratio of axonal diameter with myelin sheath and axonal diameter without myelin sheath) to assess white matter injury after ICH. Two consecutive sections from each animal at the perihematoma area were analyzed (n = 4 per group). Two images were acquired in randomly selected areas within the perihematoma from each section and analyzed with ImageJ by an investigator blinded to the experimental groups.

### 2.11 Statistical analysis

All data in this study are presented as the mean ± standard error of mean (SEM) and have been analyzed using the SPSS 22.0 software. Student's *t* test was used to analyze differences between two groups, whereas differences between multiple groups were analyzed with one‐way analysis of variance (ANOVA). Two‐way ANOVA was used to evaluate differences in the behavior tests among groups and among time points. Differences were considered significant when *P* < .05.

## 3 RESULTS

### 3.1 TGF‐β/mitogen‐activated protein kinase (MAPK) signaling mediates white matter injury by modulating inflammation after ICH in piglets

**Figure 1:**
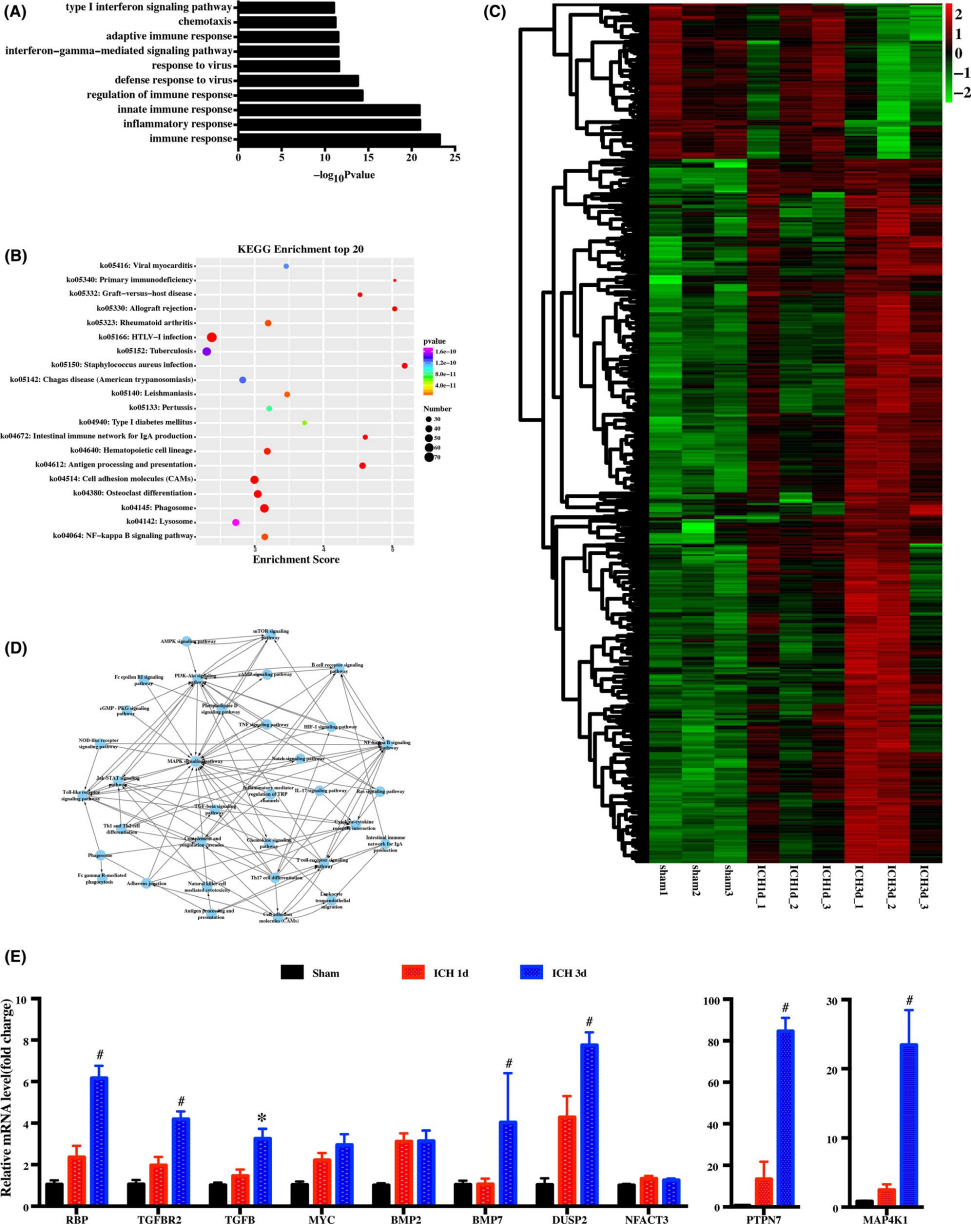
Temporal transcriptional analysis of neuroinflammation after intracerebral hemorrhage (ICH). Gene ontology (GO) biological process enrichment analysis (top 10 biological processes) (A) and pathway enrichment analysis (top 20 biological processes) (B) of genetic variations between day 3 after ICH and sham groups. C, Heatmap of the immune and inflammatory genes expressed in sham, day 1, and day 3 after ICH. D, Interaction network diagram (pathway‐pathway network) showing the signaling pathway of immunity and inflammation. E, Real‐time polymerase chain reaction (RT‐PCR) of genes in the TGF‐β and MAPK signaling pathways after ICH. n = 4 per group. *P < .05, #*P* < .01, vs sham group

To understand the changes in neuroinflammatory gene expression in white matter after ICH, we performed whole transcriptome sequencing for perihematomal white matter sorted from pig brains over the course of 3 days. Compared with the sham group, there are more than 2000 differentially expressed genes in the ICH group at day 3 postoperation. Gene ontology (GO) and pathway analyses indicate that ICH has a significant influence on the expression of genes relating to neuroinflammation (Figure 1A,B). The heatmap shows increased expression of inflammation‐related genes at day 1 and day 3 after ICH (Figure 1C). To elucidate the potential mechanisms of ICH‐induced inflammatory reactions, we performed further pathway‐pathway network analysis. This analysis identified TGF‐β and mitogen‐activated protein kinase (MAPK) pathways in the inflammatory interactions after ICH (Figure 1D). The RNA‐sequencing results were consistent with the RT‐PCR analysis of the expression of genes related to the TGF‐β and MAPK pathways (Figure 1E).

### 3.2 Treatment with minocycline causes less ICH‐induced brain swelling in piglets

**Figure 2:**
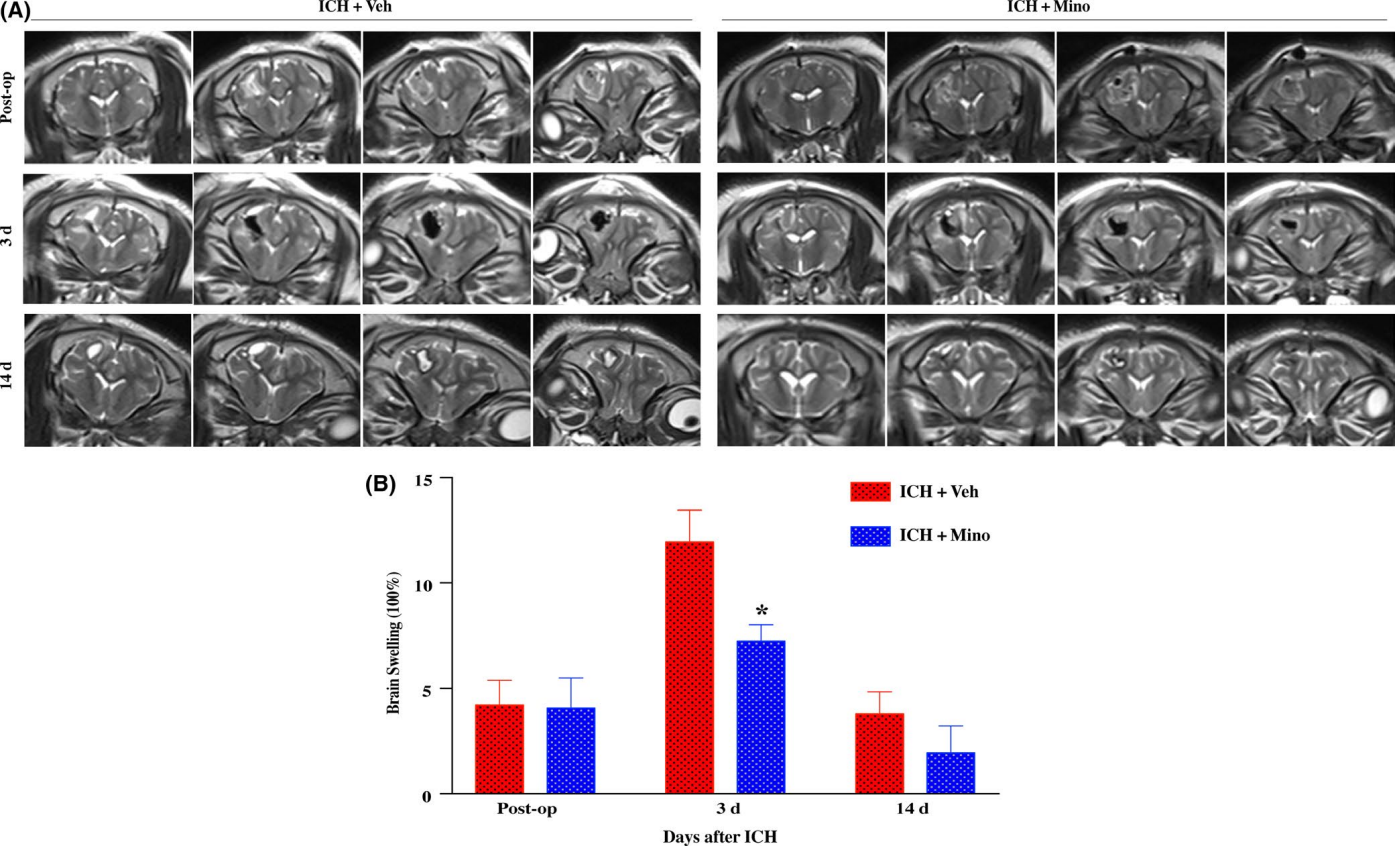
Minocycline caused less ICH‐induced brain swelling in piglets. A, Representative MRI scanning images of brain swelling changes at days 0, 3, and 14 postoperation. B, Minocycline treatment decreased brain swelling at day 3 after ICH compared with vehicle group. n = 4‐6 per group. *P < .05, #*P* < .01, vs vehicle group

T2‐weighted MRI was used to determine ICH‐induced brain swelling in piglets (Figure 2A). A blinded observer outlined the hemisphere for each animal and measured its volume. Intracerebral hemorrhage caused brain swelling in piglets at days 0, 3, and 14 compared with sham‐treated piglets (eg, day 3:11.9 ± 4.35 vs 7.23 ± 0.25, *P* < .05; Figure 2B). There was no significant difference in brain swelling with or without minocycline treatment immediately after ICH. However, treatment with minocycline results in less severe brain swelling at day 3 (11.9 ± 4.35 vs 7.23 ± 0.25 in minocycline group, *P* < .05; Figure 2B). In addition, the difference in the brain swelling ratio turned negative at day 14.

### 3.3 Treatment with minocycline causes fewer neurological deficits in piglets

**Figure 3:**
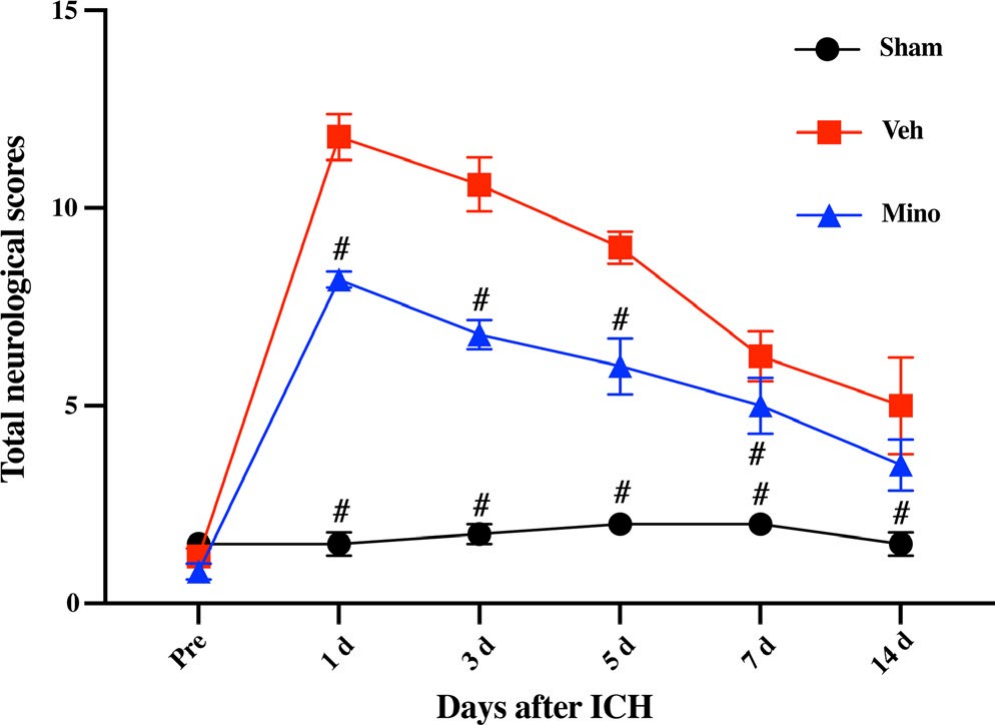
Minocycline improved neurological function deficits after intracerebral hemorrhage (ICH). Behavioral test was evaluated according to the neurological grading score with some modification, demonstrating that minocycline significantly alleviated neurological function deficits at days 1, 3, 5, and 7 after ICH, compared with vehicle group; n = 4‐5 per group. #*P* < .01 vs vehicle group

Intracerebral hemorrhage resulted in significant neurological deficits as assessed by the modified neurological grade scoring system at days 1, 3, 5, 7, and 14 after ICH (eg, day 3:1.75 ± 0.5 vs 10.6 ± 1.52, *P* < .01). However, pigs treated with minocycline had fewer neurological deficits based on their modified neurological grade score until day 7 (eg, day 7:6.8 ± 0.84 vs 10.6 ± 1.52, *P* < .01; Figure 3).

### 3.4 Treatment with minocycline causes less microglial activation in white matter after ICH in piglets

**Figure 4:**
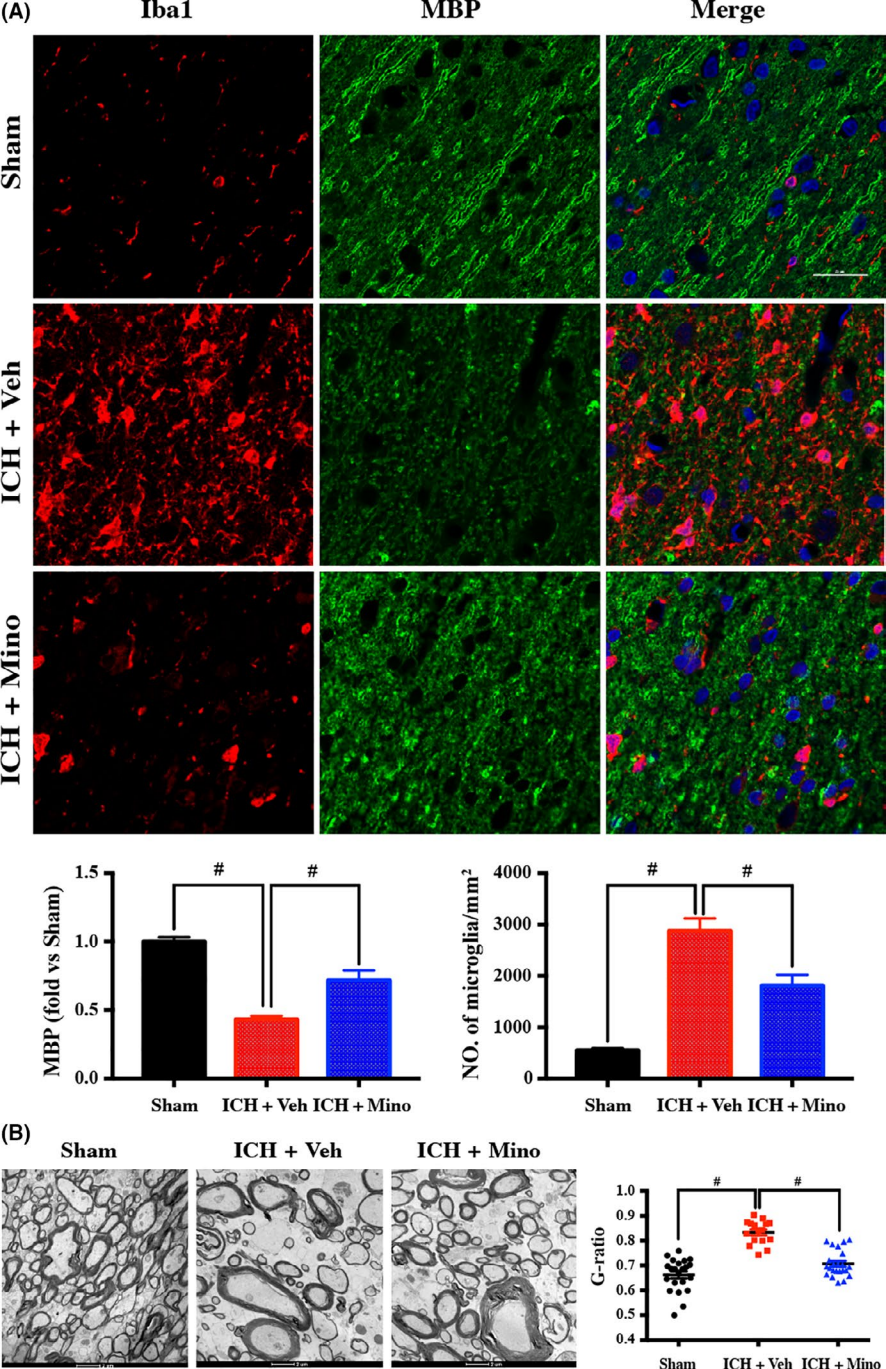
Minocycline caused less microglial activation in white matter after ICH in piglets. A, Double‐immunostaining images of microglia marker Iba1 and white matter marker MBP demonstrated that minocycline caused less microglial activation and reduced white matter injury at day 14 after ICH. B, Electron microscopy confirmed minocycline alleviated white matter at day 14 after ICH. Scale bar = 25 μm; n = 4 per group. #*P* < .01 vs vehicle group

To investigate whether minocycline affects neuroinflammation in WM, microglial activation in WM was examined at day 3. Intracerebral hemorrhage induced microglial activation and myelin basic protein (MBP) loss in WM. There were more ameboid‐shaped ionized calcium‐binding adaptor molecule 1 (Iba‐1)–positive cells in the perihematomal WM bundles 3 days after ICH. There was a significant reduction in MBP along the WM. Minocycline reduced Iba‐1 activation and alleviated the MBP reduction (Figure 4A). Electron microscopy showed demyelination of WM regions in ICH piglets, but minocycline treatment preserved the myelin sheaths due to either prevention of demyelination or induction of remyelination at 14 days post‐ICH (Figure 4B). G‐ratio (diameter of axon/whole fiber diameter) increased in the vehicle group, but not in ICH pigs treated with minocycline. These data suggest that minocycline induces remyelination after ICH.

### 3.5 Treatment with minocycline causes TGF‐β upregulation in alternatively activated microglia/macrophages in WM

**Figure 5:**
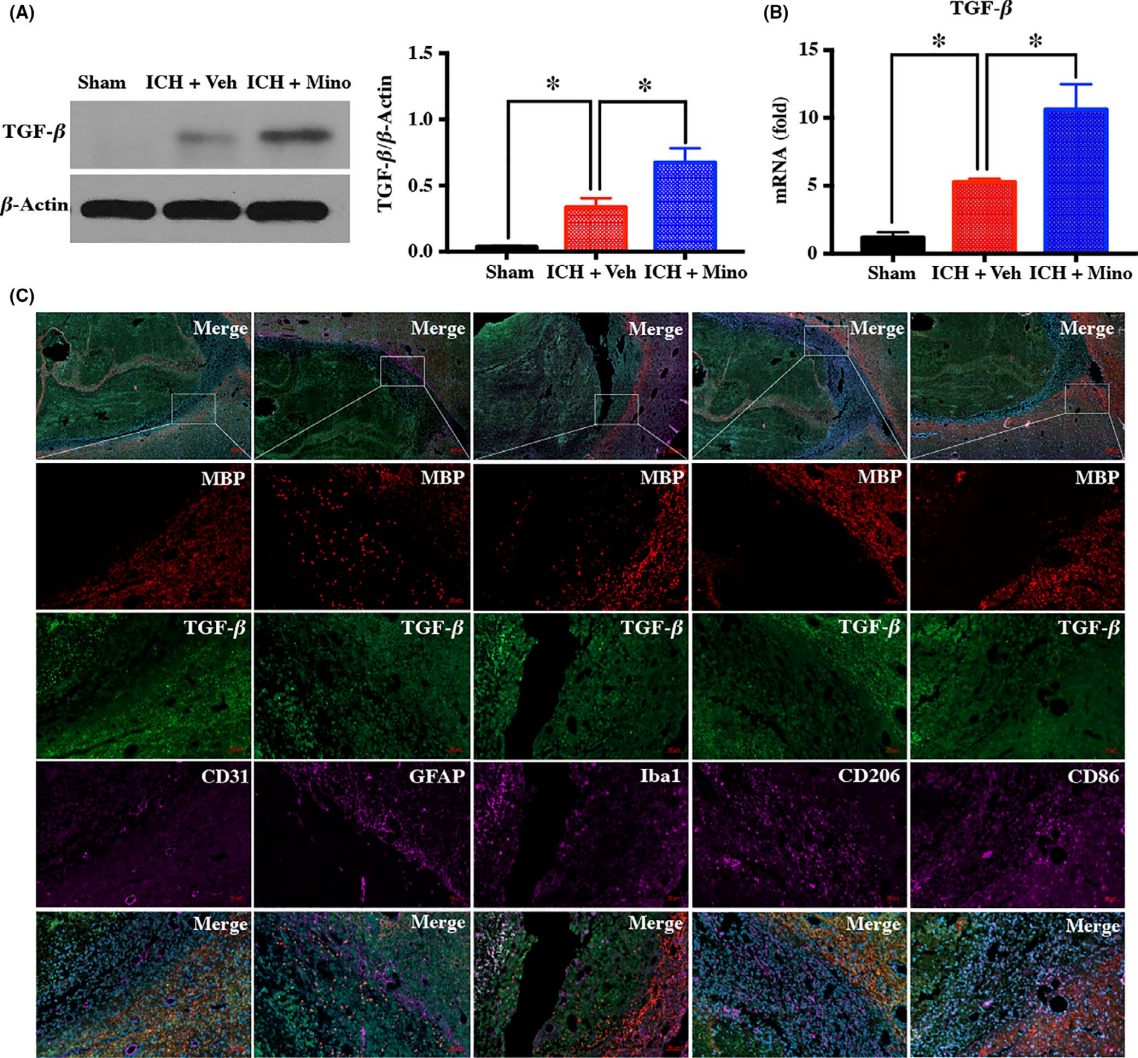
Minocycline caused transforming growth factor‐β (TGF‐β) upregulation in alternatively activated microglia/macrophages in white matter (WM). A, B, Western blot and RT‐PCR demonstrating that minocycline increased the expression of TGF‐β after intracerebral hemorrhage (ICH); n = 4 per group. C, Double‐labeling of TGF‐β with Iba‐1 (a microglia/macrophage marker), GFAP (an astrocyte marker), CD31 (an endothelial cell marker), CD206 (a M2 microglia/macrophage marker), and CD86 (a M1 microglia/macrophage marker) in white matter at day 3 after ICH. Scare bar = 50 μm; n = 3 per group. *P < .05 vs vehicle group

Given the role of minocycline in ICH‐induced WM injury, the possible effects of minocycline on the TGF‐β/MAPK signaling pathway were examined. Both RT‐PCR and Western blot confirmed that ICH caused TGF‐β upregulation in WM in comparison with the sham‐treated group. After minocycline treatment, both TGF‐β RNA and protein levels significantly increased (Figure 5A‐B). Double‐labeling showed that TGF‐β immunoreactivity colocalized with Iba‐1, but not with GFAP (an astrocyte marker) or CD31 (an endothelial cell marker). In addition, double‐labeling indicated that TGF‐β immunoreactivity colocalized with M2 microglia/macrophage marker CD206, but not with M1 microglia/macrophage marker CD86 (Figure 5C).

### 3.6 Minocycline‐induced TGF‐β upregulation suppresses neuroinflammation by modulating the MAPK signaling pathway

**Figure 6:**
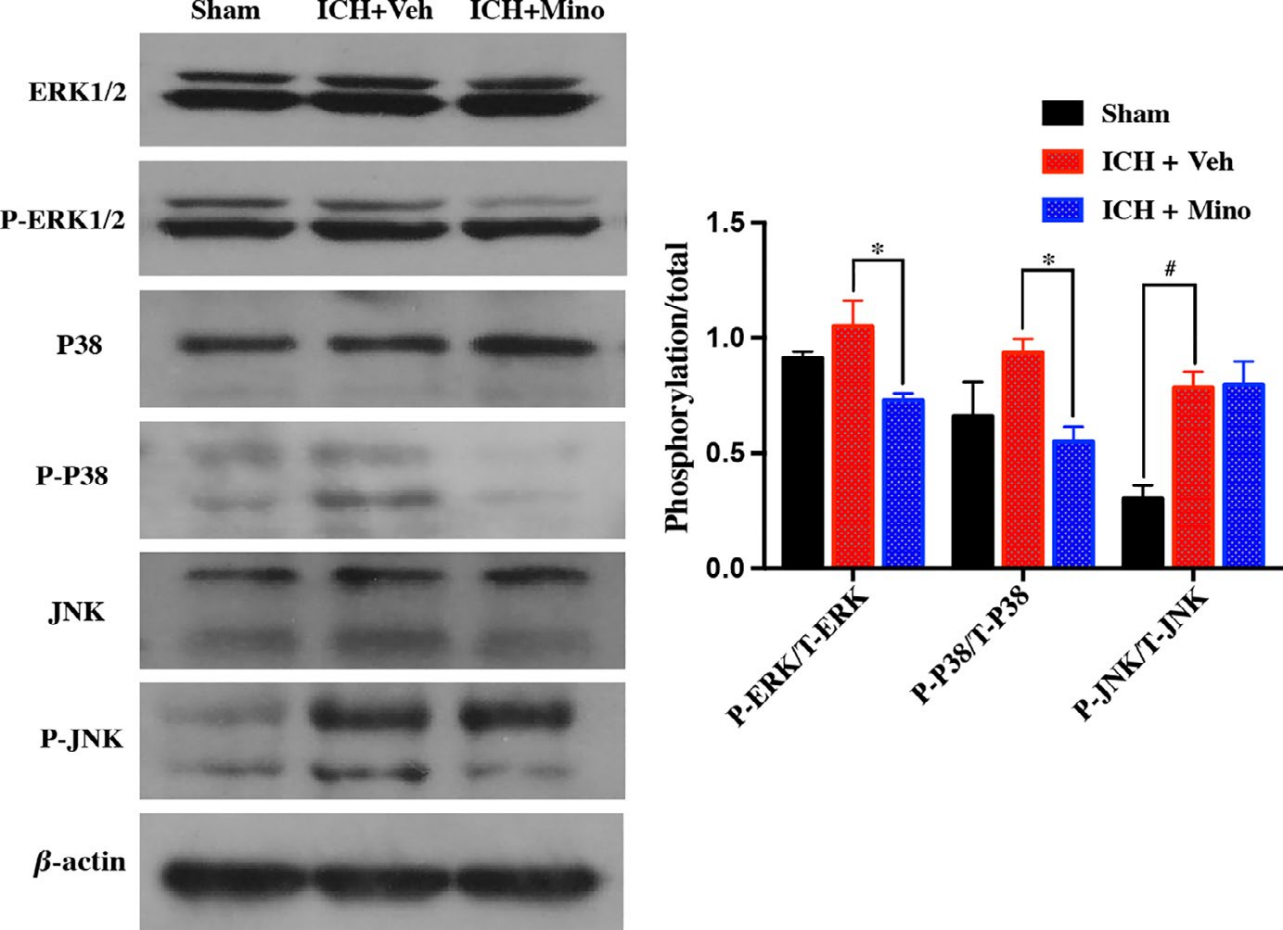
Minocycline‐induced transforming growth factor‐β (TGF‐β) upregulation modulated neuroinflammation by activating the mitogen‐activated protein kinase (MAPK) signaling pathway. A Western blot showing significant differences in protein levels of MAP kinases (extracellular signal–regulated kinase (ERK), p38, JNK, ERK, p‐p38, and p‐ERK) in various groups; n = 4 per group. *P < .05, #*P* < .01, vs vehicle group

To analyze the pathway minocycline affects during ICH‐induced WM injury, we examined the activation of ERK1/2, P38, and JNK in WM after ICH. Western blot assay confirmed that the ratio of phosphorylated/total ERK1/2, p38, and JNK increased after ICH. Treatment with minocycline decreases the activation of ERK1/2 and p38 but does not affect the activation of JNK (Figure 6).

### 3.7 Treatment with minocycline reduced iNOS, TNF‐α, and IL‐1β upregulation after ICH

**Figure 7:**
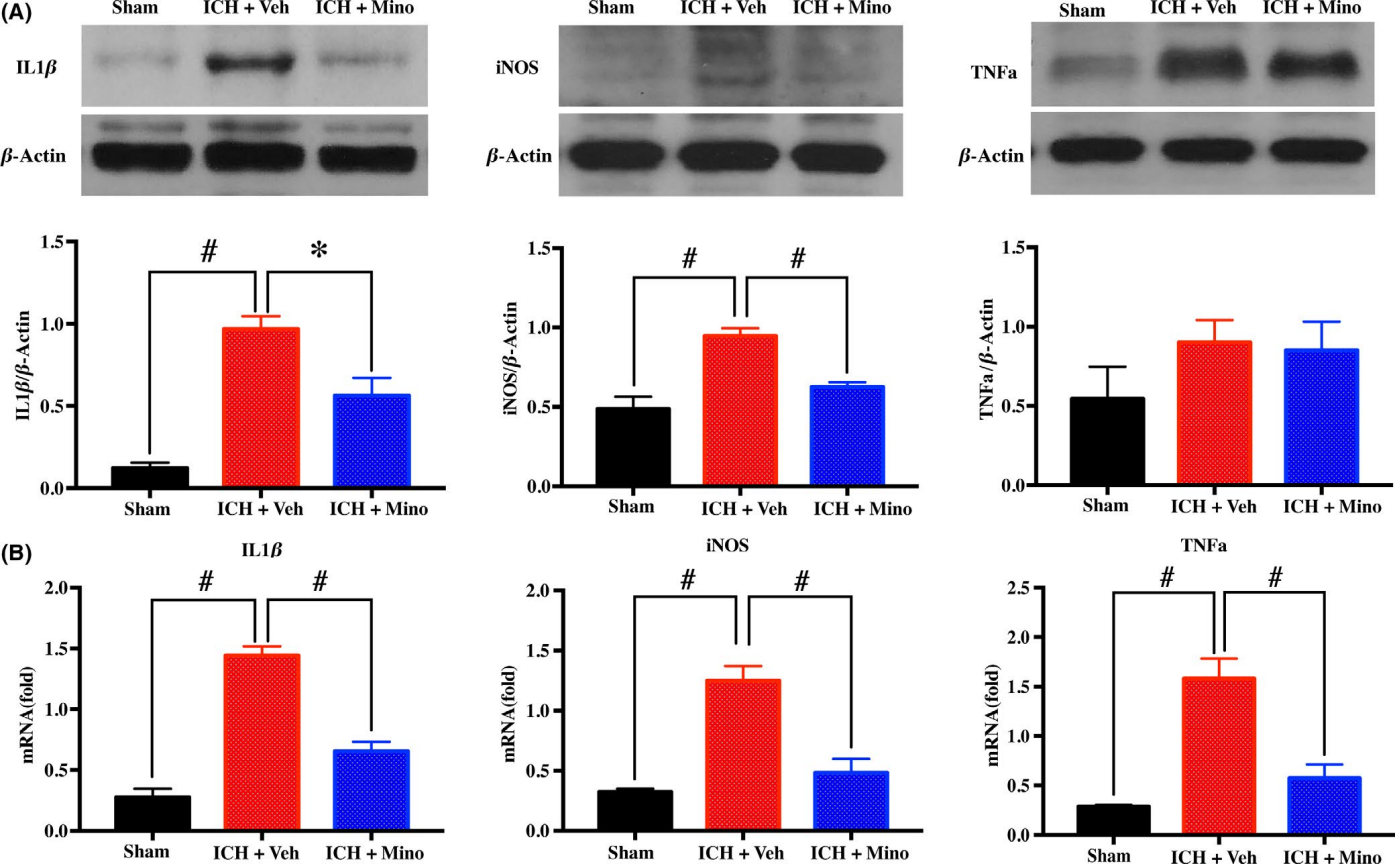
Minocycline treatment reduced neuroinflammation after intracerebral hemorrhage (ICH). A, B, Western blot and real‐time polymerase chain reaction (RT‐PCR) showing minocycline treatment significantly reduced the level of inducible nitric oxide synthase (iNOS), tumor necrosis factor‐α (TNF‐α), and interleukin‐1 beta (IL‐1β) after ICH; n = 4 per group. *P < .05, #*P* < .01, vs vehicle group

As downstream products of the MAPK pathway, iNOS, TNF‐α, and IL‐1 were examined. The effect of minocycline treatment on iNOS, TNF‐α, and IL‐1 was noticeable on day 3. Western blot or RT‐PCR showed that expression of iNOS, TNF‐α, and IL‐1 increased in white matter after ICH. Minocycline treatment significantly reduces the level of iNOS, TNF‐α, and IL‐1 after ICH (Figure 7A‐B).

## 4 DISCUSSION

In this study, we evaluated the effect of minocycline treatment on WM injury after ICH in piglets. We show that: (a) WM injury occurred after ICH and was associated with inflammation regulated by the TGF‐β/MAPK signaling pathway; (b) systemic treatment with minocycline alleviated ICH‐induced brain swelling and neurological deficits; (c) minocycline treatment caused less microglial activation, but more TGF‐β upregulation in alternatively activated microglia/macrophages in WM after ICH; and (d) minocycline treatment attenuated WM injury by triggering the TGF‐β/MAPK signaling pathway.

The effect of minocycline treatment in ICH has focused primarily on its function against iron overload in the brain. Although this function is extremely important in hemorrhagic contexts, we have discovered another mechanism for minocycline for treating secondary brain injury.^14^, ^15^ It has been shown previously that minocycline shows anti‐inflammatory, antiapoptotic, and neuroprotective effects in many models of cerebrovascular insult. Brain neuroinflammation occurs after experimental ICH and causes secondary brain injury, including brain edema, neuronal death, brain atrophy, and poor neurological outcomes.^3^ WM injury, known to be a frequent complication of ICH, is one consequence of the inflammatory response induced by blood components and metabolites.^5^ The present study showed that minocycline confers protection against hemorrhagic WM injury in a mammal model and discovered a potential mechanism for this protection via the TGF‐β/MAPK signaling pathway. These results suggest that minocycline may be ideal for use in ICH treatment.

Mitogen‐activated protein kinase subtypes, consisting of p38, JNK, and ERK1/2, are critical signaling molecules that modulate inflammation induced by several stimulators.^29^, ^30^ Our previous studies have demonstrated that ICH activated the JNK signaling pathway in WM bundles in rats. Deferoxamine, an iron chelator, reduced ICH‐induced JNK activation and white matter loss.^7^ Other reports demonstrated that suppressing P38 MAPK activation confers neuroprotection in rat intracerebral hemorrhage.^31^, ^32^ In the present study, we show through transcriptional profiling that the TGF‐β and MAPK pathways contribute significantly to inflammation and WM injury in piglets. This response was upregulated on day 1 and persisted till day 3.

The role of TGF‐β after hemorrhagic events has been elucidated previously.^33^, ^34^ Similarly, we observed a significant increase in TGF‐β levels in WM bundles after ICH in piglets. The level of TGF‐β reflects microglial polarization after ICH. M1/M2 macrophage balance polarization is known to govern the fate of neuroinflammation and brain injury after ICH.^35^, ^36^ Alternatively activated or M2 microglia/macrophages, which are polarized by cytokines such as IL‐4 and IL‐13, produce anti‐inflammatory cytokines such as IL‐10 and TGF‐β for neuroprotection, including inflammation suppression, tissue repair, remodeling, and vasculogenesis. Previous studies have shown that minocycline promotes microglial polarization via upregulation of the TrkB/BDNF pathway.^37^ Minocycline significantly reduces the levels of TNF‐α and IL‐1β, and increases the levels of TGF‐β, IL‐10, and YM‐1 in acute stroke models.^38^ Our current study reveals the changes in TGF‐β expression after ICH with or without treatment with minocycline, which may underlie minocycline's ability to reduce WM injury by activating M2 polarization via upregulating TGF‐β.

Inflammation plays a key role in white matter lesions induced by cerebral ischemia or brain trauma.^39^, ^40^ Based on our findings of microglial activation and increased expression of proinflammatory mediators in WM bundles of ICH pig models, neuroinflammation has been considered a key pathophysiology of WM injury after ICH. In addition to its microglial regulating properties, minocycline is likely also a non‐selective inhibitor of MAPKs.^41^ The current study demonstrates that minocycline inhibits the activation of p38 and extracellular signal‐regulated kinase1/2 (ERK1/2), which is supported by previous studies.^42^, ^43^, ^44^ The MAPK pathway has a key role in ICH‐induced WM injury. The current study demonstrates that inhibition of MAPK causes less WM damage, brain swelling, and neurological deficits in ICH pigs. Previous findings have shown that axonal/WM damage is associated with MAPK activation after ischemic stroke and inhibition of this kinase improves axonal/WM recovery.^45^, ^46^ WM constitutes approximately 50% of human brain volume and an even higher percentage in piglets, and WM injury can cause sensorimotor impairment, cognitive dysfunction, psychiatric disorders, gait disturbance, disequilibrium, and pain—contributing to critical neurological deficits.^47^ The present study demonstrates WM‐protective and neurorestorative roles for minocycline after ICH, according to histological, MRI, and behavioral criteria. The salutary effects of minocycline on WM integrity confirmed by electron microscopy likely contributed to the improvements in cognitive and sensorimotor functions after ICH. These results reveal a role for minocycline in the MAPK pathway's protection and repair of WM and long‐term functional recovery after ICH.

The TGF‐β‐TAK1‐MAPK pathway is a classical TGF‐β signaling pathway. TGF‐β superfamily ligands bind to type I or type II TGF‐β receptors, which recruit and phosphorylate receptors, increasing the expression of phosphorylated ERK1/2, p38, and JNK.^48^ The current study indicates that minocycline causes upregulation of TGF‐β after ICH, which decreases the activation of ERK1/2 and p38. Therefore, further research regarding the relationship TGF‐β and MAPKs after ICH is warranted.

## 5 CONCLUSIONS

In conclusion, ICH leads to acute MAPK activation in white matter in piglets. Treatment with minocycline after ICH suppresses MAPK activation by upregulating TGF‐β and attenuates WM injury.

## CONFLICT OF INTEREST

The authors declare no conflict of interest.

## Funding information

This work was financially supported by grants 81500987, 81870917, 817712374, and 81801155 from the National Natural Science Foundation of China (NSFC), grants 2014CB541604, 2016YFC1301702, and 2016YFSF110141 from National Ministry of Science and Technology (MOST), grant 15140902300 from Science and Technology Commission of Shanghai Municipality (STCSM), grant 2017BR022 from Shanghai Municipal Planning Commission of science and Research Fund, and grant 2016QD082 from the Scientific Research project supported by Huashan Hospital, Fudan University.

## References

[1] Xi G , Strahle J , Hua Y , Keep RF . Progress in translational research on intracerebral hemorrhage: is there an end in sight? Prog Neurogibol. 2014;115:45‐63. 10.1016/j.pneurobio.2013.09.007 PMC3961535 24139872

[2] Keep RF , Hua Y , Xi G . Intracerebral haemorrhage: mechanisms of injury and therapeutic targets. Lancet Neurol. 2012;11:720‐731. 22698888 10.1016/S1474-4422(12)70104-7 PMC3884550

[3] Xi G , Keep RF . Intracerebral hemorrhage: mechanisms and therapies. Transl Stroke Res. 2012;3:1‐3. 10.1007/s12975-012-0189-2 24323857

[4] Lee SH , Kim BJ , Ryu WS , et al. White matter lesions and poor outcome after intracerebral hemorrhage: a nationwide cohort study. Neurology. 2010;74:1502‐1510. 20458066 10.1212/WNL.0b013e3181dd425a

[5] Zuo S , Pan P , Li Q , Chen Y , Feng H . White matter injury and recovery after hypertensive intracerebral hemorrhage. Biomed Res Int. 2017;2017:6138424. 28680884 10.1155/2017/6138424 PMC5478825

[6] Rost NS , Rahman RM , Biffi A , et al. White matter hyperintensity volume is increased in small vessel stroke subtypes. Neurology. 2010;75:1670‐1677. 21060091 10.1212/WNL.0b013e3181fc279a PMC3033608

[7] Ni W , Okauchi M , Hatakeyama T , et al. Deferoxamine reduces intracerebral hemorrhage‐induced white matter damage in aged rats. Exp Neurol. 2015;272:128‐134. 25749188 10.1016/j.expneurol.2015.02.035 PMC4668331

[8] Leys D , Englund E , Del Ser T , et al. White matter changes in stroke patients. Relationship with stroke subtype and outcome. Eur Neurol. 1999;42:67‐75. 10473977 10.1159/000069414

[9] Pantoni L , Leys D , Fazekas F , et al. Role of white matter lesions in cognitive impairment of vascular origin. Alzheimer Dis Assoc Disord. 1999;13(Suppl 3):S49‐54. 10609681

[10] Taylor RA , Chang CF , Goods BA , et al. TGF‐beta1 modulates microglial phenotype and promotes recovery after intracerebral hemorrhage. J Clin Invest. 2017;127:280‐292. 27893460 10.1172/JCI88647 PMC5199690

[11] Dziewulska D , Rafalowska J . Role of endoglin and transforming growth factor‐beta in progressive white matter damage after an ischemic stroke. Neuropathology. 2006;26:298‐306. 16961065 10.1111/j.1440-1789.2006.00700.x

[12] Koistinaho M , Malm TM , Kettunen MI , et al. Minocycline protects against permanent cerebral ischemia in wild type but not in matrix metalloprotease‐9‐deficient mice. J Cereb Blood Flow Metab. 2005;25:460‐467. 15674236 10.1038/sj.jcbfm.9600040

[13] Plane JM , Shen Y , Pleasure DE , Deng W . Prospects for minocycline neuroprotection. Arch Neurol. 2010;67:1442‐1448. 20697034 10.1001/archneurol.2010.191 PMC3127230

[14] Cao S , Hua Y , Keep RF , Chaudhary N , Xi G . Minocycline effects on intracerebral hemorrhage‐induced iron overload in aged rats: brain iron quantification with magnetic resonance imaging. Stroke. 2018;49:995‐1002. 29511126 10.1161/STROKEAHA.117.019860 PMC5871578

[15] Zhao F , Hua Y , He Y , Keep RF , Xi G . Minocycline‐induced attenuation of iron overload and brain injury after experimental intracerebral hemorrhage. Stroke. 2011;42:3587‐3593. 21998050 10.1161/STROKEAHA.111.623926 PMC3226873

[16] Fagan SC , Waller JL , Nichols FT , et al. Minocycline to improve neurologic outcome in stroke (MINOS): a dose‐finding study. Stroke. 2010;41:2283‐2287. 20705929 10.1161/STROKEAHA.110.582601 PMC3916214

[17] Fouda AY , Newsome AS , Spellicy S , et al. Minocycline in acute cerebral hemorrhage: an early phase randomized trial. Stroke. 2017;48:2885‐2887. 28887388 10.1161/STROKEAHA.117.018658

[18] Jalal FY , Yang Y , Thompson JF , Roitbak T , Rosenberg GA . Hypoxia‐induced neuroinflammatory white‐matter injury reduced by minocycline in SHR/SP. J Cereb Blood Flow Metab. 2015;35:1145‐1153. 25712499 10.1038/jcbfm.2015.21 PMC4640265

[19] Ma J , Zhang J , Hou WW , et al. Early treatment of minocycline alleviates white matter and cognitive impairments after chronic cerebral hypoperfusion. Sci Rep. 2015;5:12079. 26174710 10.1038/srep12079 PMC4502604

[20] Xi G , Keep RF , Hoff JT . Mechanisms of brain injury after intracerebral haemorrhage. Lancet Neurol. 2006;5:53‐63. 16361023 10.1016/S1474-4422(05)70283-0

[21] Wagner KR , Xi G , Hua YA , et al. Lobar intracerebral hemorrhage model in pigs: rapid edema development in perihematomal white matter. Stroke. 1996;27:490‐497. 8610319 10.1161/01.str.27.3.490

[22] Xi G , Wagner KR , Keep RF , et al. Role of blood clot formation on early edema development after experimental intracerebral hemorrhage. Stroke. 1998;29:2580‐2586. 9836771 10.1161/01.str.29.12.2580

[23] Zhou X , Xie Q , Xi G , Keep RF , Hua Y . Brain CD47 expression in a swine model of intracerebral hemorrhage. Brain Res. 2014;1574:70‐76. 24931767 10.1016/j.brainres.2014.06.003 PMC4121112

[24] Gu Y , Hua Y , Keep RF , Morgenstern LB , Xi G . Deferoxamine reduces intracerebral hematoma‐induced iron accumulation and neuronal death in piglets. Stroke. 2009;40:2241‐2243. 19372448 10.1161/STROKEAHA.108.539536 PMC2693321

[25] Yamaguchi M , Zhou C , Heistad DD , Watanabe Y , Zhang JH . Gene transfer of extracellular superoxide dismutase failed to prevent cerebral vasospasm after experimental subarachnoid hemorrhage. Stroke. 2004;35:2512‐2517. 15472087 10.1161/01.STR.0000145198.07723.8e

[26] Xie Q , Gu Y , Hua Y , Liu W , Keep RF , Xi G . Deferoxamine attenuates white matter injury in a piglet intracerebral hemorrhage model. Stroke. 2014;45:290‐292. 24172580 10.1161/STROKEAHA.113.003033 PMC3886635

[27] Wang G , Zhang J , Hu X , et al. Microglia/macrophage polarization dynamics in white matter after traumatic brain injury. J Cereb Blood Flow Metab. 2013;33:1864‐1874. 23942366 10.1038/jcbfm.2013.146 PMC3851898

[28] Huang S‐Q , Tang C‐L , Sun S‐Q , et al. Demyelination initiated by oligodendrocyte apoptosis through enhancing endoplasmic reticulum‐mitochondria interactions and Id2 expression after compressed spinal cord injury in rats. CNS Neurosci Ther. 2014;20:20‐31. 23937638 10.1111/cns.12155 PMC6493115

[29] Zhao H , Wang SL , Qian L , et al. Diammonium glycyrrhizinate attenuates Abeta(1–42) ‐induced neuroinflammation and regulates MAPK and NF‐kappaB pathways in vitro and in vivo. CNS Neurosci Ther. 2013;19:117‐124. 23279783 10.1111/cns.12043 PMC6493378

[30] Pyo H , Jou I , Jung S , Hong S , Joe EH . Mitogen‐activated protein kinases activated by lipopolysaccharide and beta‐amyloid in cultured rat microglia. NeuroReport. 1998;9:871‐874. 9579682 10.1097/00001756-199803300-00020

[31] Ohnishi M , Monda A , Takemoto R , et al. Sesamin suppresses activation of microglia and p44/42 MAPK pathway, which confers neuroprotection in rat intracerebral hemorrhage. Neuroscience. 2013;232:45‐52. 23228810 10.1016/j.neuroscience.2012.11.057

[32] Cao J , Zhuang Y , Zhang J , et al. Leucine‐rich repeat kinase 2 aggravates secondary brain injury induced by intracerebral hemorrhage in rats by regulating the P38 MAPK/Drosha pathway. Neurobiol Dis. 2018;119:53‐64. 30048803 10.1016/j.nbd.2018.07.024

[33] Gomes FC , Sousa Vde O , Romao L . Emerging roles for TGF‐beta1 in nervous system development. Int J Dev Neurosci. 2005;23:413‐424. 15936920 10.1016/j.ijdevneu.2005.04.001

[34] Douglas MR , Daniel M , Lagord C , et al. High CSF transforming growth factor beta levels after subarachnoid haemorrhage: association with chronic communicating hydrocephalus. J Neurol Neurosurg Psychiatry. 2009;80:545‐550. 19066194 10.1136/jnnp.2008.155671

[35] Zhou K , Zhong Q , Wang YC , et al. Regulatory T cells ameliorate intracerebral hemorrhage‐induced inflammatory injury by modulating microglia/macrophage polarization through the IL‐10/GSK3beta/PTEN axis. J Cereb Blood Flow Metab. 2017;37:967‐979. 27174997 10.1177/0271678X16648712 PMC5363473

[36] Chen Z‐Q , Yu H , Li H‐Y , et al. Negative regulation of glial Tim‐3 inhibits the secretion of inflammatory factors and modulates microglia to antiinflammatory phenotype after experimental intracerebral hemorrhage in rats. CNS Neurosci Ther. 2019;25(6):674‐684. 30677253 10.1111/cns.13100 PMC6515709

[37] Miao H , Li R , Han C , Lu X , Zhang H . Minocycline promotes posthemorrhagic neurogenesis via M2 microglia polarization via upregulation of the TrkB/BDNF pathway in rats. J Neurophysiol. 2018;120:1307‐1317. 29790836 10.1152/jn.00234.2018

[38] Yang Y , Salayandia VM , Thompson JF , Yang LY , Estrada EY , Yang Y . Attenuation of acute stroke injury in rat brain by minocycline promotes blood‐brain barrier remodeling and alternative microglia/macrophage activation during recovery. J Neuroinflammation. 2015;12:26. 25889169 10.1186/s12974-015-0245-4 PMC4340283

[39] Pu H , Guo Y , Zhang W , et al. Omega‐3 polyunsaturated fatty acid supplementation improves neurologic recovery and attenuates white matter injury after experimental traumatic brain injury. J Cereb Blood Flow Metab. 2013;33:1474‐1484. 23801244 10.1038/jcbfm.2013.108 PMC3764381

[40] Xu MY , Wang YF , Wei PJ , Gao YQ , Zhang WT . Hypoxic preconditioning improves long‐term functional outcomes after neonatal hypoxia‐ischemic injury by restoring white matter integrity and brain development. CNS Neurosci Ther. 2019;25(6):734‐747. 30689302 10.1111/cns.13102 PMC6515700

[41] Möller T , Bard F , Bhattacharya A , et al. Critical data‐based re‐evaluation of minocycline as a putative specific microglia inhibitor. Glia. 2016;64:1788‐1794. 27246804 10.1002/glia.23007

[42] Won KA , Kang YM , Lee MK , et al. Participation of microglial p38 MAPK in formalin‐induced temporomandibular joint nociception in rats. J Orofac Pain. 2012;26:132‐141. 22558613

[43] Cho IH , Lee MJ , Jang M , Gwak NG , Lee KY , Jung HS . Minocycline markedly reduces acute visceral nociception via inhibiting neuronal ERK phosphorylation. Mol Pain. 2012;8:13. 22364340 10.1186/1744-8069-8-13 PMC3342906

[44] Nikodemova M , Duncan ID , Watters JJ . Minocycline exerts inhibitory effects on multiple mitogen‐activated protein kinases and IkappaBalpha degradation in a stimulus‐specific manner in microglia. J Neurochem. 2006;96:314‐323. 16336636 10.1111/j.1471-4159.2005.03520.x

[45] Barone FC , Irving EA , Ray AM , et al. SB 239063, a second‐generation p38 mitogen‐activated protein kinase inhibitor, reduces brain injury and neurological deficits in cerebral focal ischemia. J Pharmacol Exp Ther. 2001;296:312‐321. 11160612

[46] Legos JJ , Erhardt JA , White RF , et al. SB 239063, a novel p38 inhibitor, attenuates early neuronal injury following ischemia. Brain Res. 2001;892:70‐77. 11172750 10.1016/s0006-8993(00)03228-5

[47] Tao C , Hu X , Li H , You C . White matter injury after Intracerebral Hemorrhage: pathophysiology and therapeutic strategies. Front Hum Neurosci. 2017;11:422. 28890692 10.3389/fnhum.2017.00422 PMC5575148

[48] Guo X , Wang XF . Signaling cross‐talk between TGF‐beta/BMP and other pathways. Cell Res. 2009;19:71‐88. 19002158 10.1038/cr.2008.302 PMC3606489

