# Supplementary material for: Minocycline reduces intracerebral hemorrhage–induced white matter injury in piglets
Source: CNS Neurosci Ther. 2019 Sep 26;25(10):1195–206. doi: 10.1111/cns.13220 (PMC6776747; doi:10.1111/cns.13220)
Supplement: Supplementary file 1 [file CNS-25-1195-s001.docx]

|  | ICH+Veh | | | | | | ICH+Mino | | | | |
| --- | --- | --- | --- | --- | --- | --- | --- | --- | --- | --- | --- |
| Post-op | 6.01 | 3.68 | 8.83 | 1.80 | 3.93 | 1.06 | 9.03 | 1.34 | 1.34 | 4.81 | 3.84 |
| 3d | 17.29 | 8.86 | 12.98 | 8.42 | 9.06 | 15.01 | 9.19 | 5.99 | 4.95 | 8.45 | 7.59 |
| 14d | 5.42 | 3.38 | 6.06 | 0.17 | 1.51 | 7.93 | 4.01 | 0.91 | 0.43 | 2.06 | 4.22 |

**Supplemental table 1: The quantification data of brain water content**
